# Expanded newborn screening for inherited metabolic disorders by tandem mass spectrometry in a northern Chinese population

**DOI:** 10.3389/fgene.2022.801447

**Published:** 2022-09-30

**Authors:** Hong Zhang, Yanyun Wang, Yali Qiu, Chao Zhang

**Affiliations:** ^1^ Suqian Maternal and Child Health Care Hospital, Suqian, China; ^2^ Nanjing Maternal and Child Health Care Hospital, Nanjing, China

**Keywords:** newborn screening, inborn errors, tandem mass spectrometry, inherited metabolic disorders, next-generation sequencing

## Abstract

Tandem mass spectrometry (MS/MS) has been developed as one of the most important diagnostic platforms for the early detection and screening of inherited metabolic disorders (IMDs). To determine the disease spectrum and genetic characteristics of IMDs in Suqian city of Jiangsu province in the northern Chinese population, dried blood spots from 2,04,604 newborns, were assessed for IMDs by MS/MS from January 2016 to November 2020. Suspected positive patients were diagnosed through next-generation sequencing (NGS) and validated by Sanger sequencing. One hundred patients with IMDs were diagnosed, resulting in an overall incidence of 1/2,046, of which 56 (1/3,653), 22 (1/9,300), and 22 (1/9,300) were confirmed amino acids disorders (AAs), organic acids disorders (OAs), fatty acid oxidation disorders (FAODs) positive cases, respectively. The highest incidence of IMDs is phenylalanine hydroxylase deficiency (PAHD) (45 cases), with a total incidence of 1:4,546. Hot spot mutations in phenylalanine hydroxylase (PAH)-related genes are *c.158G > A* (24.44%), *c.728G > A* (16.67%), *c.611A > G* (7.78%), and *c.331C>T* (7.78%). The related hot spot mutation of the *MMACHC* gene is *c.609G > A* (45.45%). Short-chain acyl-CoA dehydrogenase deficiency (SCAD)-related *ACADS* gene hotspot mutations are *c.164C > T* (33.33%) and *c.1031A > G* (33.33%). Our work indicated that the overall incidence of IMDs is high, and the mutations in *PAH*, *ACADS*, and *MMACHC* genes are the leading causes of IMDs in Suqian city. The incidence of AAs in Suqian city is higher than in other Chinese areas. The disease spectrum and genetic backgrounds were elucidated, contributing to the treatment and prenatal genetic counseling of these disorders in this region.

## Introduction

Inherited metabolic disorders (IMDs) or inborn errors of metabolism (IEM) are a class of metabolic disorders caused by gene mutations, representing roughly 1,000 different genetic disorders (Ferreira et al., 2019). Even though they are individually rare, their collective prevalence is estimated today at greater than 1:800 individuals (Wilcox, 2018). The key to clinical management of IMDs is to obtain a definitive genetic diagnosis followed by clinical interventions, such as dietary modification, metabolite administration, or enzyme replacement therapy, to reduce mortality and morbidity and improve quality of life (Tarailo-Graovac et al., 2016). Newborn screening (NBS) is a valuable preventive health measure for early diagnosis, which is diagnostically effective and economically efficient (Zhang et al., 2021).

Tandem mass spectrometry (MS/MS) has been developed as a diagnostic platform for early detection, and screening of genetic disorders and many countries have implemented NBS using MS/MS (Garg and Dasouki, 2006), especially for AAs, OAs, FAODs (Chace et al., 2003; Fabie et al., 2019). The incidence of IMDs detected by MS/MS varies significantly among different countries. The incidence rate of IMDs was 1:8,557 in Japan, 1:7,030 in Taiwan, and 1:2,200 in Germany (Shibata et al., 2018). Already in 2012, Shi et al. have been reported that the average incidence rate of IMDs was 1/3,795 using the NBS of MS/MS in mainland china (Shi et al., 2012a).

In recent years, more and more regions of China, such as Shanghai, Guangzhou, Zhejiang, and Guangxi, have implemented NBS using MS/MS and reported IMDs incidence in their area (Huang et al., 2012; Guo et al., 2018; Wang et al., 2019; Zhang et al., 2021). Substantial progress in disease prevention, saving lives, and improving patient prognosis has been made in China since screening for IMDs in newborns (Lin et al., 2019; Wang et al., 2019).

In China, children with no clinical symptoms are diagnosed and treated through MS/MS screening technology, reducing the disability rate and mortality. In addition, it has significantly reduced the economic burdens on the family and society (Shi et al., 2012a; Ye et al., 2015; Chen et al., 2018; Wang et al., 2019). With the soaring development of genomics and molecular biology, next-generation sequencing (NGS) has become the gold standard and a common tool used for the diagnostic evaluation of IMDs (Leonard and Morris, 2006; Dai et al., 2019). Focusing on 204,604 newborns, we aimed to determine the disease spectrum and genetic characteristics of IMDs in Suqian city and explore the application value of NBS for IMDs using MS/MS.

## Materials and methods

### Subjects

2,04,604 infants born in Suqian city were enrolled for expanded NBS by MS/MS from January 2016 to November 2020. The Ethical Committee of Suqian Maternity and Children’s Hospital approved this study. Written informed consents were obtained from all the infants’ patients.

### NBS flow chart

**FIGURE 1:**
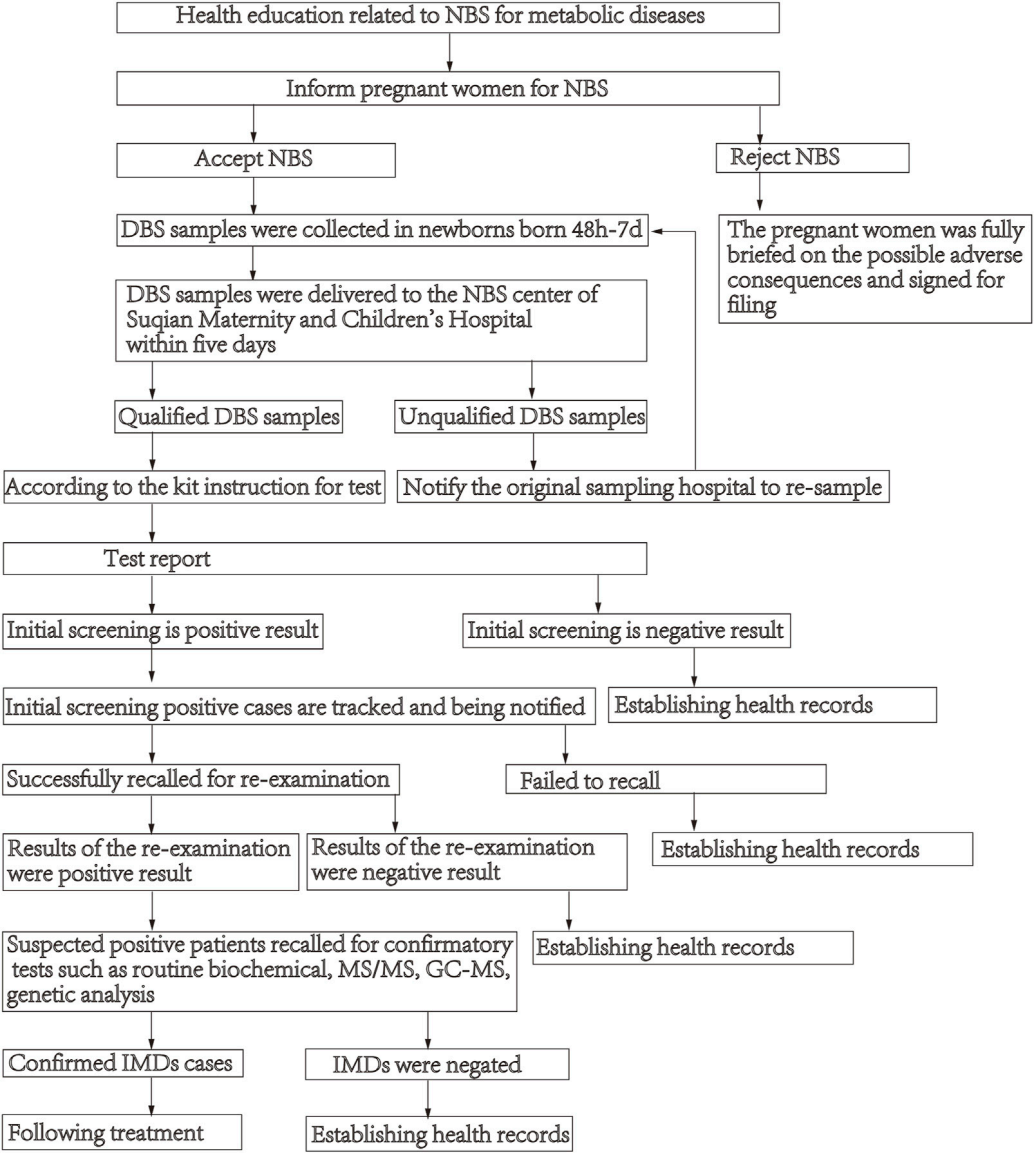
Flowchart of newborn screening.

Dried blood spots (DBS) samples were collected following standard procedures to collect DBS in newborns born 48 h-7 days after lactation. DBS samples were delivered by cold-chain transportation to the NBS center of Suqian Maternity and Children’s Hospital within 5 days, and then they were analyzed using MS/MS. If the test result is higher or lower than the cut-off value, the initial screening is positive, and the child is recalled for re-examination. Those with abnormal results (suspected positive patients) are recalled for confirmatory tests such as routine biochemical, MS/MS, gas-chromatographic mass spectrometry (GC-MS) and genetic analysis. The description of the process of NBS see Figure 1.

### Mass spectrometry analysis

DBS were pre-processed following the instruction of NeoBaseTM non-derivatized MS/MS kit (PerkinElmer, MA, United States), using 1525u high-performance liquid chromatography (HPLC) (Waters Technologies, Milford, MA, United States) and ACQUITY TQD mass spectrometer (Waters, Milford, MA, USA) for quantitative analysis. The analytes included 11 amino acids, 31 acylcarnitine, and 1 Ketone (succinylacetone). The 11 amino acids were alanine (Ala), arginine (Arg), citrulline (Cit), glycine (Gly), leucine/isoleucine (Leu/lle/Pro-OH), methionine (Met), ornithine (Orn), phenylalanine (Phe), proline (Pro), tyrosine (Tyr), and valine (Val); the 31 acylcarnitine were free carnitine (C0), acetylcarnitine (C2), propionylcarnitine (C3), malonylcarnitine/3-hydroxy- butyrylcarnitine (C3DC/C4OH), butyrylcarnitine (C4), methylmalonyl/3-hydroxy- isovalerylcarnitine (C4DC/C5OH), isovalerylcarnitine (C5), tiglylcarnitine (C5:1), glutarylcarnitine/3-Hydroxy-hexanoylcarnitine (C5DC/C6OH), hexanoylcarnitine (C6), adipylcarnitine (C6DC), octanoylcarnitine (C8), octenoylcarnitine (C8:1), decanoylcarnitine (C10), decenoylcarnitine (C10:1), decadienoylcarnitine (C10:2), dodecanoylcarnitine (C12), dodecenoylcarnitine (C12:1), tetradecanoylcarnitine (C14), tetradecenoylcarnitine (C14:1), tetradecadienoylcarnitine (C14:2), 3-Hydroxy-tetradecanoylcarnitine (C14OH), hexadecanoylcarnitine (C16), hexadecenoylcarnitine (C16:1), 3-Hydroxy-hexadecanoylcarnitine (C16OH), 4-Hydroxy-hexadecenoylcarnitine (C16:1OH), octadecanoylcarnitine (C18), octadecenoylcarnitine (C18:1), octadecadienoylcarnitine (C18:2), 3-Hydroxy-Octadecanoylcarnitine (C18OH), 3-Hydroxy-Octadecenoylcarnitine (C18:1OH).

The indoor quality control and the inter-room quality control adopt the quality evaluation standard of NBS by the Clinical Examination Center of the Ministry of Health, and they are all qualified. The cut-off values were initially set by reference to the worldwide collaborative project and other screening centers (Niu et al., 2010; McHugh et al., 2011) and were adjusted over time as the number of samples increased.

### Next-generation sequencing analysis

Genetic analysis was performed by Genuine Diagnostics Company (Hangzhou, Zhejiang, China). The detailed process of NGS was as follows. According to the manufacturer’s protocol, DBS or peripheral whole blood of suspected positive patients was referred to a laboratory, and genomic DNA was extracted using the QIA amp DNA Blood Midi Kit (Qiagen, Hilden, Germany). Then use Gene Cap gene sequence capture technology to mix standard IMDs pathogenic gene capture probes with the child’s whole genome library, and the disease-related gene fragments are hybridized for the examination, and the DNA in the non-target area is eluted. The fragments are washed away, thereby enriching the pathogenic gene fragments. cDNA library was constructed and sequenced using Illumina HiSeq 2000 sequencer (San Diego, CA, United States). After the fragments were filtered and trimmed by the Trim-Galore program, the sequences with reading quality >20 and read length >80 bp are retained. The sequenced reads were aligned to the human reference genome (hg19) using the Burrows-Wheeler Aligner (BWA). Then, the GATH software package was used to collect point mutations, insertion mutations or deletion mutations, etc. Next, all variants identified by NGS were further validated by Sanger sequencing of the parents. In addition, multiple ligation-dependent probe amplification (MLPA) technology is also used to diagnose diseases where NGS has no pathogenic mutations but suspected deletion or duplication mutations, such as non-ketotic hyperglycinemia and propionic acidemia (PA).

### Diagnosis and follow-up

Metabolic disease specialists made a definitive diagnosis based on the patients’ biochemical performance, genetic mutations, and clinical symptoms. Then, a definitive diagnosis was made by metabolic disease specialists based on the patients’ biochemical performance, genetic mutations, and clinical symptoms. Only patients diagnosed by the genetic analysis were included in this study. All infants with negative screening results were included in the children’s health care management system for follow-up. All confirmed children were followed up every 3–6 months, and the follow-up data were collected.

## Results

### Newborn screening

**FIGURE 2:**
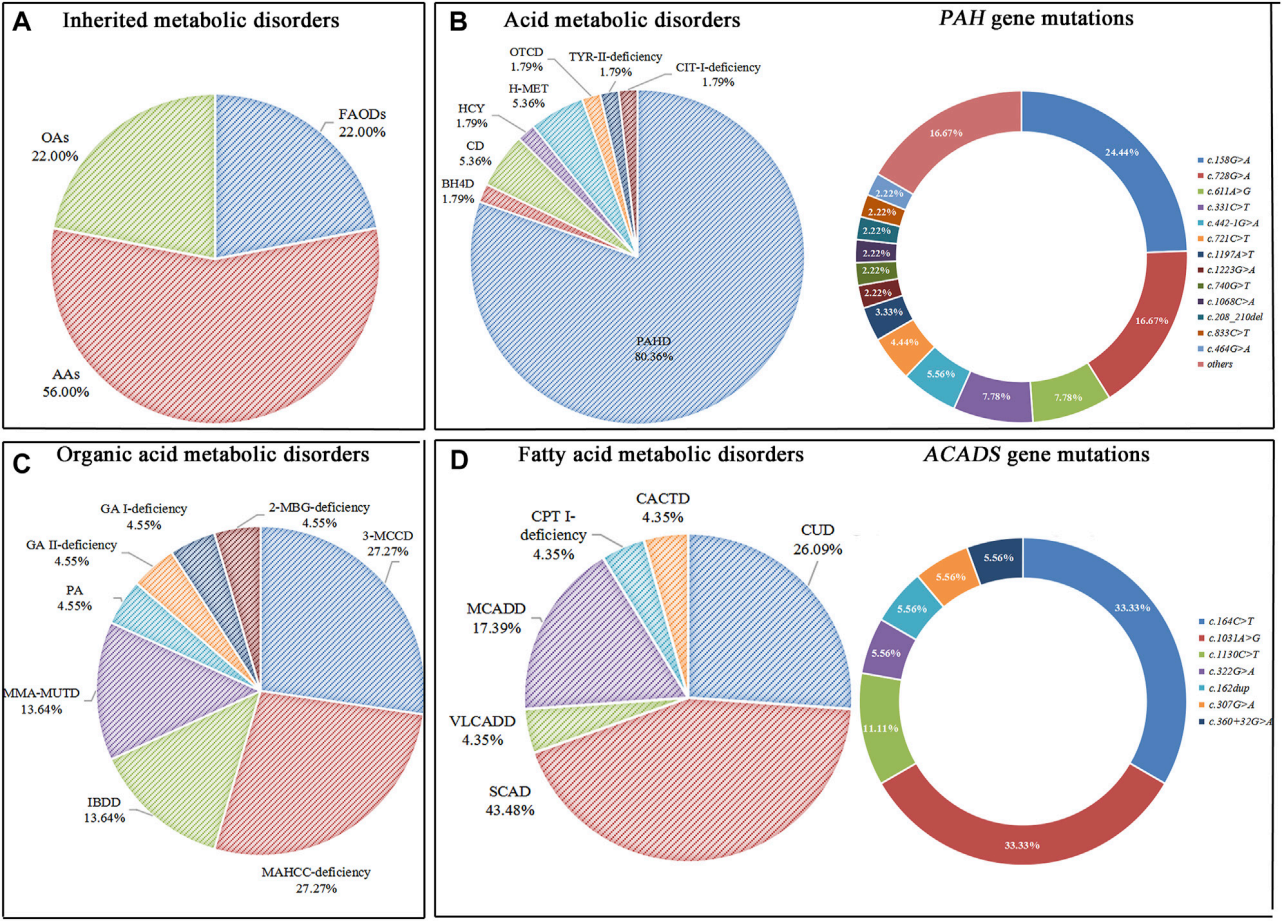
Disease spectrum, distribution, and gene mutations of inherited metabolic disorders. **(A)** The percentage of three categories of inherited metabolic disorders. **(B)** The percentage of different types of amino acid metabolic disorders and alleles of *PAH* gene mutations. **(C)** The percentage of different types of organic acid metabolic disorders. **(D)** The percentage of different types of fatty acid metabolic disorders and alleles of *ACADS* gene mutations.

An expanded NBS program screened 2,04,604 newborns. After initial screening, 4,069 (1.99%) newborns, who had positive results, were recalled for a new specimen. 4,021 newborns (98.82%) were successfully recalled and had a repeated test, 100 cases were finally confirmed with IMDs, and the positive predictive value (PPV) was 2.46%. Twenty-two types of IMDs were diagnosed in 100 confirmed cases, and the overall IMDs detection incidence was 1: 2,046; of these, 56 (56.0%) newborns with AAs, 22 (22.0%) with OAs, and 22 (22.0%) with FAODs. AAs, OAs, and FAODs were 1:3,653, 1:9,300, and 1:9,300, respectively. Details are shown in Table 1 and Figure 2A.

**TABLE 1:** Abnormal MS/MS markers and results statistics of all confirmed infants.

Disorders	Positive cases	Frequency	Abnormal MS/MS marks	Concentration mean (range) (μmol/L)	Reference range (μmol/L)
Amino acids disorders (AAs)	56	1:3653			
Phenylalanine hydroxylase deficiency (PAHD)	45	1:4546	Phe	413.99 (108.57–1631.90)	24–105
			Phe/Tyr	7.52 (1.14–32.95)	0.16–1.25
Tetrahydrobiopterin deficiency (BH4D)	1	1:204604	Phe	267.42	24–105
			Phe/Tyr	3.67	0.16–1.25
Citrin deficiency (CD)	3	1:68201	Cit	116.66 (48.54–218.23)	5.5–30
Homocysteinemia (HCY)	1	1:204604	Met	100.16	6–40
Hypermethioninemia (H-MET)	3	1:68201	Met	205.82 (59.55–296.34)	6–40
			Met/Phe	5.96 (5.12–6.80)	0.12–0.73
Ornithine transcarbamylase deficiency (OTCD)	1	1:204604	Cit	5.45	5.5–30
			Cit/Phe	0.12	0.13–0.89
Tyrosine aminotransferase type II deficiency (TYR II-deficiency)	1	1:204604	Tyr	666.75	35–320
Citrullinemia type I deficiency (CIT I-deficiency)	1	1:204604	Cit	135.68	5.5–30
			Cit/Phe	2.857	0.10–0.75
Organic acids disorders (OAs)	22	1:9300			
Propionic acidemia (PA)	1	1:204604	C3	4.68	0.3–0.45
			C3/C2	0.33	0.01–0.2
3-methylcrotonyl CoA carboxylase deficiency (3-MCCD)	6	1:34100	C4DC/C5OH	3.15 (0.81–8.82)	0.08–0.4
			(C4DC/C5OH)/C0	0.06 (0.04–0.09)	0–0.02
			(C4DC/C5OH)/C8	31.97 (16.50–51.75)	1.2–15
Methylmalonic aciduria and homocystinuria type C deficiency (MAHCC-deficiency)	6	1:34100	C3	6.08 (4.32–8.24)	0.3–0.45
			C3/C0	0.35 (0.15–0.55)	0.02–0.2
			C3/C2	0.82 (0.35–1.95)	0.01–0.2
Isobutyryl-CoA dehydrogenase deficiency (IBDD)	3	1:68201	C4	1.29 (0.73–1.98)	0.08–0.45
			C4/C2	0.13 (0.07–0.17)	0–0.03
			C4/C3	1.00 (0.86–1.09)	0.04–0.39
Methylmalonic aciduria mut type deficiency (MMA-MUTD)	3	1:68201	C3	11.27 (8.28–16.58)	0.3–0.45
			C3/C2	0.54 (0.42–0.67)	0.01–0.2
Glutaric acidemia type II deficiency (GA II-deficiency)	1	1:204604	C4	0.64	0.08–0.45
			C6	0.27	0.01–0.09
			C8	0.46	0.01–0.13
			C10	0.37	0.02–0.21
			C5DC/C6OH	0.49	0.04–0.2
Glutaric acidemia type I deficiency (GA I-deficiency)	1	1:204604	C0	5.52	9.5–65
			C5DC/C6OH	2.01	0.04–0.2
			(C5DC/C6OH)/(C3DC/C4OH)	33.5	0.35–2.33
			(C5DC/C6OH)/(C4DC/C5OH)	9.14	0–1.14
2-methylbutyrylglycinuria deficiency (2-MBG-deficiency)	1	1:204604	C5	0.66	0.03–0.35
			C5/C2	0.02	0–0.04
			C5/C3	0.52	0.02–0.42
Fatty acid oxidation disorders (FAODs)	22	1:9300			
Carnitine uptake defect (CUD)	6	1:34100	C0	5.45 (3.66–6.95)	9–50
Short-chain acyl-CoA dehydrogenase deficiency (SCAD)	11	1:18600	C4	0.99 (0.62–1.46)	0.08–0.45
			C4/C2	0.07 (0.03–0.10)	0–0.03
			C4/C3	0.72 (0.33–1.14)	0.04–0.39
Very long-chain acyl-CoA dehydrogenase deficiency (VLCADD)	1	1:204604	C14:1	3.06	0.02–0.26
Medium-chain acyl-CoA dehydrogenase deficiency (MCADD)	2	1:102302	C6	0.29 (0.28–0.31)	0.01–0.09
			C8	0.80 (0.61–1.00)	0.01–0.15
			C10	0.26 (0.2–0.31)	0.02–0.2
			C8/C2	0.09	0–0.01
Carnitine palmitoyl transferase I deficiency (CPT I-deficiency)	1	1:204604	C0	70.83	9–50
Carnitine-acylcarnitine translocase deficiency (CACTD)	1	1:204604	C0/(C16/C18)	43.19	2.4–35
			C6	0.63	0.01–0.09

Note: Phe, phenylalanine; Tyr, tyrosine; Cit, citrulline; Met, methionine; C2, aceylcarnitine; C3, propionylcarnitine; C5DC, glutarylcarnitine; C5OH, 3-hydroxyisovalerylcarnitine/3-hydroxy-2-methylbutyrylcarnitine; C0, free carnitine; C8, octanoylacrnitine; C10, decanoylcarnitine; C12, dodecanoylcarnitine; C14, myristoylcarnitine; The results did not exceed the reference ranges including amino acids were alanine (Ala), arginine (Arg), glycine (Gly), leucine/isoleucine (Leu/lle/Pro-OH), ornithine (Orn), proline (Pro), valine (Val), tiglylcarnitine (C5:1), adipylcarnitine (C6DC), octenoylcarnitine (C8:1), decenoylcarnitine (C10:1), decadienoylcarnitine (C10:2), dodecanoylcarnitine (C12), dodecenoylcarnitine (C12:1), tetradecanoylcarnitine (C14), tetradecadienoylcarnitine (C14:2), 3-Hydroxy-tetradecanoylcarnitine (C14OH), hexadecanoylcarnitine (C16), hexadecenoylcarnitine (C16:1), 3-Hydroxy-hexadecanoylcarnitine (C16OH), 4-Hydroxy-hexadecenoylcarnitine (C16:1OH), octadecanoylcarnitine (C18), octadecenoylcarnitine (C18:1), octadecadienoylcarnitine (C18:2), 3-Hydroxy-Octadecanoylcarnitine (C18OH), 3-Hydroxy-Octadecenoylcarnitine (C18:1OH).

### Amino acids disorders

Totally eight types of AAs were detected. Phenylalanine hydroxylase deficiency (PAHD) was the most common disorder (45/56, 80.4%), followed by citrin deficiency (CD) (3/56, 5.36%), hypermethioninemia (H-MET, 3/56, 5.36%). There was only one positive case for tetrahydrobiopterin deficiency (BH4D), homocysteinemia (HCY), ornithine transcarbamylase deficiency (OTCD), and tyrosine aminotransferase type II deficiency (TYR II- deficiency). The phenylalanine concentration of 45 PAHD patients exceeded the reference range (reference range: 24–105 μmol/L), and only 3 patients’ phenylalanine/tyrosine ratios were within the reference range (reference range: 0.16–1.25 μmol/L). The phenylalanine concentration and phenylalanine/tyrosine ratios of the 3 patients were 120.69, 113.44, 141.12 μmol/L, and 1.168, 1.244, 1.14, respectively. Details are shown in Table 1.

Forty-five patients had *PAH* gene mutations, of whom two patients had concurrent *PTS* gene and *PAH* gene mutations. The most frequent mutation found was *c.158G>A* (22/90, 24.44%), followed by *c.728G>A* (15/90, 16.67%), *c.611A>G* (7/90, 7.78%), *c.331C>T* (7/90, 7.78%). Three patients with CD were female, and mutations were compound heterozygous. The *MAT1A* mutations of two patients with H-MET were heterozygous, and the mutations were *c.777_778insCG* and *c.895C>T*. One patient has a compound heterozygous *c.1003T>C* and *c.188G>T*. Details are shown in Table 1 and Figure 2B. (Supplementary File S1: Supplementary Table S1).

### Organic acids disorders

There were eight types of OAs. Of these types, the most common disorder was 3-methylcrotonyl CoA carboxylase deficiency (3-MCCD, 6/22, 27.27%) and methylmalonic aciduria and homocystinuria type C deficiency (MAHCC- deficiency, 6/22, 27.27%), followed by isobutyryl-CoA dehydrogenase deficiency (IBDD, 3/22, 13.63%), methylmalonic aciduria mut type deficiency (MMA-MUTD, 3/22, 13.63%). The remaining four types of OAs were all of one case. Details are shown in Table 1.

Six patients with 3-MCCD found a total of 13 site mutations in 5 mutated genes, of whom three patients were compound heterozygous, and one was heterozygous in the *MCCC2* gene, two patients were heterozygous in the *MCCC1* gene. Six patients with MAHCC deficiency had *MAHCC* gene mutations, and the most common mutation was *c.609G>A* (5/11, 45.45%). In patients with MMA-MUTD, the predominant mutation in *MUT* gene was *c.323G>A* and *c.729_730insTT*, with each frequency of 33.33%. Three patients with IBDD have compound heterozygous mutations in the *ACAD8* gene and one with mutations in the *PAH* gene. The remaining patients were either compound heterozygous mutation, and no high frequent mutation was found. Details are shown in Table 1 and Figure 2C. (Supplementary file S2: Supplementary Table S2).

### Fatty acid oxidation disorders

Six types of FAODs were detected among the 22 cases. SCAD was the most common disease in this group, which accounted for 50.00%, followed by CUD (6/22, 27.27%), and medium-chain acyl-CoA dehydrogenase deficiency (MCADD, 2/22, 9.09%). The very long-chain acyl- CoA dehydrogenase deficiency (VLCADD), carnitine palmitoyl transferase I deficiency (CPT I-deficiency), and carnitine-acylcarnitine translocase deficiency (CACTD) were comparatively rare. In the initial screening, one case was recalled due to CIT (CIT = 73.55; C0 = 70.83, C0/(C16 + C18) = 43.19). However, Next-generation sequencing detected *PAH* gene *c.473G>A* locus heterozygous mutation, *CPT1A* gene *c.1910C>T* and *c.1065G>A* locus heterozygous mutation, and finally diagnosed as CPT-I deficiency. Details are shown in Table 1.

One case was recalled due to an abnormal concentration of C5 in the initial screening. However, Next-generation sequencing detected *ACADM* gene *c.1085G>A* locus heterozygous mutation, *ACADSB* gene *c.655G>A* and *c.848A>G* locus heterozygous mutation, and finally diagnosed as SCAD. There were six types of FAODs and 27 mutation sites in 25 genes. The most mutation gene was *ACADS* (10/25, 40.00%), and the most common mutation was *c.1031A>G* (6/18, 33.33%) and *c.164C>T* (6/18, 33.33%). Other mutation sites were comparatively rare. Details are shown in Table 1 and Figure 2D. (Supplementary File S3: Supplementary Table S3).

### Follow-up in the confirmed cases

The average initial screening time was 14.39 ± 6.59 days, and the second screening was 66.8 ± 148.11 days. Two patients died, one due to recurrent fever, poor response, hypoketotic hypoglycemia, and liver injury (period 21 months), while another was unknown (period 6 months). One patient with MMA-MUTD had normal intelligence but presented with hypotonia and developmental delays. Exception for three patients (one each in MAHCC-deficiency, MMA-MUTD, and PA) whose parents refused to follow up, the remaining 94 children received regular follow-up, and all are generally developing at present. The longest follow-up period is approximately 6 years (Supplementary File S4: Supplementary Table S4)

## Discussion

The overall incidence of IMDs detected by MS/MS in northern Chinese, Suqian city is 1: 2,046. Several articles reported the overall incidence of IMDs in other regions of Chinese, for example, northwestern Chinese 1:1,898 (Zhang et al., 2021), eastern China 1:1,178 (Guo et al., 2018), southern Chinese 1:2,804 (18). The overall incidence of IMDs in other countries appeared to be lower than that of Chinese, for example, 1:8,557 in Japan (Shibata et al., 2018), 1:7,030 in Taiwan (Shibata et al., 2018), 1:13,205 in South Korea (Shibata et al., 2018), 1:2,200 in Germany (Shibata et al., 2018), 1:4,942 in the Faroe Islands and Greenland (Lund et al., 2012), 1: 6,000 in European (Smon et al., 2018). The possible cause of the difference is the influence of the government policies on NBS. Nearly all babies in China are tested at birth for rare, serious, and treatable disorders through mandatory province NBS. Another possible reason is that it did not use any second-tier testing regarding MS/MS screening in China (Zhang et al., 2021). The results of this study showed that the AAs were the most common style of IMDs, accounting for 56.00% of patients (1:3,653), followed by OAs (22.00%; 1:9,300) and FAODs (22.00%; 1:9,300). For other Chinese areas, the incidence of AAs, OAs and FAODs was 1:4,176, 1:5,220, 1:12,179 in northwestern Chinese (Zhang et al., 2021), 1:5,084, 1:13,389, 1:9,129 in Suzhou (Wang et al., 2019), 1:8680, 1:9347 and 1:7440 respectively in southern Chinese (Lin et al., 2019). This means that the incidence of AAs in Suqian city is higher than in other Chinese areas.

PAHD, SCAD, CUD, 3-MCCD, and MAHCC-deficiency are the most common IMDs, and hotspot mutations in pathogenic genes are consistent with Suzhou and the Southern region in China (Lin et al., 2019; Wang et al., 2019). 4,021 suspected positive patients were using MS/MS diagnosis, NGS confirmed 100 cases and the PPV was 2.49%. It shows that MS/MS has a certain degree of false-positive rate when used in screening IMDs. On the one hand, false positives are related to the limitations of MS/MS. For example, the test results are easily affected by factors such as gestational age, newborn weight, and nutritional status. On the other hand, it is related to metabolism, such as abnormal liver and kidney function, drug treatment, diet, and non-metabolic diseases that cause secondary or transient metabolic disorders. Xia et al. (2021) found that vitamin B12 deficiency and acidosis in pregnant women can cause an increase in plasma C3 in their newborns. Arnold et al. (Arnold et al., 2008) reported that the mild secondary increase in 3-hydroxyisovalerylcarnitine (C5OH) in healthy newborns was due to the mother’s 3-methylcrotonyl-coenzyme glycinuria. In 2012, Shi et al. (Shi et al., 2012a) reported on NBS for IMDs in mainland China for the previous 30 years. There were only 371,942 neonates screened in Mainland China by MS/MS from 2008 to 2012. However, there are 204,604 infants born in Suqian city enrolled for expanded NBS using MS/MS from January 2016 to November 2020. These substantial changes may be attributed to the efforts of the Chinese government policy and financial support.

In this study, 56 patients with AAs were confirmed, accounting for 56.0% of patients with IMDs, and the incidence rate was 1:3,467. The incidence of PAHD is the highest among all IMDs, reaching 1:4,546. It is higher than the incidence of PAHD in the Chinese population reported in the literature (total incidence: 1/11,614) (Shi et al., 2012b). According to the concentration of blood phenylalanine, the Chinese Preventive Medicine Association classified PAH deficiency into three types, including mild hyperphenylalaninemia (MHP) (120–360 μmol/L), mild phenylketonuria (mPKU) (360–1,200 μmol/L), classic phenylketonuria (cPKU) (≥1,200 μmol/L) (Association et al., 2019). MHP accounted for 57.78% (26/45) in the present study, and cPKU accounted for 4.44% (2/45) in patients with PAH deficiency. Previous studies show that MHP and mPKU have a higher incidence in the Chinese population than cPKU, which is more prevalent in Eastern Europe (Chen et al., 2018). In the present study, MHP and mPKU accounted for 95.56% of patients with PAHD, consistent with the reports in the literature. This study found 89 different *PAH* gene mutations, of which two cases had mutations in the *PAH* gene and *PTS* gene. The most common mutations were *c.158G>A* (23.5%) and *c.728G>A* (16.8%). It is a hotspot mutation in the Chinese population, and it has also been confirmed that PAHD has a variety of variants and genotypes in different people, and the phenotype is complex (Lin et al., 2019).

In summary, we propose the following conclusions. The advantage of MS/MS is its shorter detection time, super sensitivity, and specificity, making it a powerful tool for screening for IMDs in newborns. The disease occurs of IMDs the Suqian region has the following characteristics. IMDs are not rare in the Suqian region, particularly for AAs. In this region, recurrent mutations of relatively common diseases like PAHD, SCAD, CUD, 3-MCCD, and MAHCC-deficiency were also elucidated. The NBS strategy of combining MS/MS with NGS can improve the early diagnosis of IMDs and facilitate necessary interventions.

There are some limitations of this study. Concerning M/SMS screening efficiency, like most newborn screening centers in China, we did not use any second-tier testing, so we had a high positive rate of initial screening. Moreover, a bias in sample acquisition may exist due to the samples only from hospitals qualified for NBS in Suqian. Therefore, our results may not accurately reflect the urban and rural distribution of IMDs in the Suqian area. Besides, this study could not provide detailed information on the routine biochemistry of patients and their mothers (Bower et al., 2019; Hazan et al., 2020; Raskind and El-Chaar, 2000; Scriver et al., 2001; Tebani et al., 2016; The People’s Government of Jiangsu Province, 2019).

## Data Availability

The data presented in the study are deposited in the MetaboLights database repository, accession number MTBLS5673.

## Abbreviations

IMDs inherited metabolic disorders; NBS newborn screening; MS/MStandem mass spectrometry; NGS next-generation sequencing; AAs amino acids disorders; OAs organic acids disorders; FAODs fatty acid oxidation disorders; MLPA multiplex ligation-dependent probe amplification; PA propionic acidemia; DBS dried blood spots; PPV positive predictive value; PAHD phenylalanine hydroxylase deficiency; BH4D tetrahydrobiopterin deficiency; CD citrin deficiency; HCY homocysteinemia; H-MET hypermethioninemia; OTCD ornithine transcarbamylase deficiency; TYR-II tyrosine aminotransferase type II; CIT-I citrullinemia type I; CUD carnitine uptake defect; SCAD short-chain acyl-CoA dehydrogenase deficiency; VLCADD very long-chain acyl-CoA dehydrogenase deficiency; MCADD medium-chain acyl-CoA dehydrogenase deficiency; CPT-I carnitine palmitoyl transferase-I; CACTD carnitine-acylcarnitine translocase deficiency; 3-MCCD 3-methylcrotonyl; CoA carboxylase deficiency; MAHCC methylmalonic aciduria and homocystinuria cblC type; IBDD isobutyryl-CoA dehydrogenase deficiency; MMA-MUTD methylmalonic aciduria mut type deficiency; GA-II glutaric acidemia type II; GA-I glutaric acidemia type I; 2-MBG 2-methylbutyrylglycinuria; C2 aceylcarnitine; C3 propionylcarnitine; C5 isovalerylcarnitine/2-methylbutyrylcarnitine; C5DC glutarylcarnitine; C5OH 3-hydroxyisovalerylcarnitine/3-hydroxy-2-methylbutyrylcarnitine; C0 free carnitine; C8 octanoylacrnitine; C10 decanoylcarnitine; C12 dodecanoylcarnitine; C14 myristoylcarnitine.
