# Supplementary material for: Expanded newborn screening for inherited metabolic disorders by tandem mass spectrometry in a northern Chinese population
Source: Front Genet. 2022 Sep 30;13:801447. doi: 10.3389/fgene.2022.801447 (PMC9562093; doi:10.3389/fgene.2022.801447)
Supplement: Supplementary file 1 [file Table2.DOCX]

**Table S1** Molecular diagnosis of 56 patients with amino acid disorders

| No. | Gender | Disorders | Affected Gene | Allele 1 | Allele 2 |
| --- | --- | --- | --- | --- | --- |
| 1 | Female | Tetrahydrobiopterin deficiency | *PTS* | *c.84_291A>G* | *c.286G>A* |
| 2 | Male | Phenylalanine hydroxylase deficiency | *PAH* | *c.116_118del* | *c.728G>A* |
| 3 | Female | Citrin deficiency | *SLC25A13* | *c.124delA* | *c.1177G>A* |
| 4 | Female | Phenylalanine hydroxylase deficiency | *PAH* | *c.158G>A* | *c.833C>T* |
| 5 | Female | Phenylalanine hydroxylase deficiency | *PAH* | *c.158G>A* | *c.331C>T* |
| 6 | Female | Phenylalanine hydroxylase deficiency | *PAH* | *c.158G>A* | *c.611A>G* |
| 7 | Male | Phenylalanine hydroxylase deficiency | *PAH* | *c.158G>A* | *c.611A>G* |
| 8 | Male | Phenylalanine hydroxylase deficiency | *PAH* | *c.158G>A* | *c.1238G>C* |
| 9 | Female | Phenylalanine hydroxylase deficiency | *PAH* | *c.158G>A* | *c.611A>G* |
| 10 | Female | Phenylalanine hydroxylase deficiency | *PAH* | *c.158G>A* | *c.728G>A* |
| 11 | Female | Phenylalanine hydroxylase deficiency | *PAH* | *c.158G>A* | *c.728G>A* |
| 12 | Female | Phenylalanine hydroxylase deficiency | *PAH* | *c.158G>A* | *c.208_210del* |
| 13 | Male | Phenylalanine hydroxylase deficiency | *PAH* | *c.158G>A* | *c.442-1G>A* |
| 14 | Male | Phenylalanine hydroxylase deficiency | *PAH* | *c.158G>A* | *c.331C>T* |
| 15 | Female | Phenylalanine hydroxylase deficiency | *PAH* | *c.158G>A* | *c.442-1G>A* |
| 16 | Female | Phenylalanine hydroxylase deficiency | *PAH* | *c.158G>A* | *c.1301C>A* |
| 17 | Male | Phenylalanine hydroxylase deficiency | *PAH* | *c.158G>A* | *c.1197A>T* |
| 18 | Male | Phenylalanine hydroxylase deficiency | *PAH* | *c.158G>A* | *c.842+2T>C* |
| 19 | Male | Phenylalanine hydroxylase deficiency | *PAH* | *c.158G>A* | *c.1068C>A* |
| 20 | Female | Phenylalanine hydroxylase deficiency | *PAH* | *c.158G>A* | *c.1222C>T* |
| 21 | Male | Phenylalanine hydroxylase deficiency | *PAH* | *c.158G>A* | *c.1223G>A* |
| 22 | Male | Phenylalanine hydroxylase deficiency | *PAH* | *c.158G>A* | *c.722G>A* |
| 23 | Female | Phenylalanine hydroxylase deficiency | *PAH* | *c.158G>A* | *c.728G>A* |
| 24 | Male | Phenylalanine hydroxylase deficiency | *PAH* | *c.158G>A* | *c.208_210del* |
| 25 | Male | Phenylalanine hydroxylase deficiency | *PAH* | *c.158G>A* | *c.913-7A>G* |
| 26 | Female | Hypermethioninemia | *MAT1A* | *c.188G>T* | *c.1003T>C* |
| 27 | Male | Ornithine transcarbamylase deficiency | *OTC* | *c.317G>C* |  |
|  |  |  | *MMACHC* | *c.482G>A* |  |
| 28 | Male | Phenylalanine hydroxylase deficiency | *PAH* | *c.331C>T* | *c.1223G>A* |
| 29 | Male | Phenylalanine hydroxylase deficiency | *PAH* | *c.331C>T* | *c.721C>T* |
| 30 | Female | Phenylalanine hydroxylase deficiency | *PAH* | *c.331C>T* | *c.1197A>T* |
| 31 | Female | Phenylalanine hydroxylase deficiency | *PAH* | *c.331C>T* | *c.728G>A* |
| 32 | Female | Phenylalanine hydroxylase deficiency | *PAH* | *c.331C>T* | *c.1123C>G* |
| 33 | Male | Homocysteinemia | *CBS* | *c.374G>A* | *c.1126G>A* |
|  |  |  | *CPS1* | *c.711+1G>A* |  |
| 34 | Female | Phenylalanine hydroxylase deficiency | *PAH* | *c.442-1G>A* | *c.728G>A* |
| 35 | Female | Phenylalanine hydroxylase deficiency | *PAH* | *c.442-1G>A* | *c.728G>A* |
| 36 | Male | Phenylalanine hydroxylase deficiency | *PAH* | *c.442-1G>A* | *c.611A>G* |
| 37 | Male | Phenylalanine hydroxylase deficiency | *PAH* | *c.464G>A* | *c.728G>A* |
| 38 | Female | Phenylalanine hydroxylase deficiency | *PAH* | *c.464G>A* | *c.1194A>G* |
| 39 | Male | Phenylalanine hydroxylase deficiency | *PAH* | *c.611A>G* | *c.740G>T* |
| 40 | Female | Phenylalanine hydroxylase deficiency | *PAH* | *c.611A>G* |  |
| 41 | Male | Phenylalanine hydroxylase deficiency | *PAH* | *c.611A>G* | *c.833C>T* |
| 42 | Female | Phenylalanine hydroxylase deficiency | *PAH* | *c.721C>T* | *c.728G>A* |
| 43 | Male | Phenylalanine hydroxylase deficiency | *PAH* | *c.721C>T* | *c.728G>A* |
| 44 | Male | Phenylalanine hydroxylase deficiency | *PAH* | *c.721C>T* | *c.728G>A* |
| 45 | Male | Phenylalanine hydroxylase deficiency | *PAH* | *c.728G>A* |  |
| 46 | Male | Phenylalanine hydroxylase deficiency | *PAH* | *c.728G>A* | *c.740G>T* |
| 47 | Male | Phenylalanine hydroxylase deficiency | *PAH* | *c.728G>A* | *c.1068C>A* |
| 48 | Male | Phenylalanine hydroxylase deficiency | *PAH* | *c.728G>A* | *c.1197A>T* |
| 49 | Male | Citrullinemia type I | *ASS1* | *c.773+4A>C* | *c.1168G>A* |
| 50 | Male | Hypermethioninemia | *MAT1A* | *c.777_778insCG* |  |
| 51 | Female | Tyrosine aminotransferase type II | *TAT* | *c.821C>A* | *c.1297C>T* |
| 52 | Female | Citrin deficiency | *SLC25A13* | *c.852_855del* | *IVS16ins3kb* |
| 53 | Female | Hypermethioninemia | *MAT1A* | *c.895C>T* |  |
| 54 | Female | Phenylalanine hydroxylase deficiency | *PAH* | *c.913-2del* | *c.1315+6T>A* |
|  |  |  | *PTS* | *c.259C>T* |  |
| 55 | Male | Phenylalanine hydroxylase deficiency | *PAH* | *c.977G>A* | *c.332G>A* |
| 56 | Female | Citrin deficiency | *SLC25A13* | *c.1177+1G>A* | *IVS16ins3kb* |
