# Supplementary material for: Expanded newborn screening for inherited metabolic disorders by tandem mass spectrometry in a northern Chinese population
Source: Front Genet. 2022 Sep 30;13:801447. doi: 10.3389/fgene.2022.801447 (PMC9562093; doi:10.3389/fgene.2022.801447)
Supplement: Supplementary file 2 [file Table5.DOCX]

**Table S4** The follow-up of 100 patients identified with inherited metabolic disorders

| **No.** | **Diagnosis** | **Types of disorders** | **Age at initial screening (days)** | **Age at the second screening (days)** | **current symptoms** | **Prognosis** |
| --- | --- | --- | --- | --- | --- | --- |
| 1 | HCY | AAs | 13 | 35 | Normal intelligence, normal physique | Under treatment |
| 2 | H-MET | AAs | 9 | 23 | Low methionine diet; normal intelligence, normal physique | Under treatment |
| 3 | CD | AAs | 10 | 18 | Milk powder product fortified with medium-chain triglyceride and restricted with galactose; normal intelligence, normal physique | Under treatment |
| 4 | CD | AAs | 17 | 30 | Milk powder product fortified with medium-chain triglyceride and restricted with galactose; normal intelligence, normal physique | Under treatment |
| 5 | CD | AAs | 19 | 37 | Milk powder product fortified with medium-chain triglyceride and restricted with galactose; normal intelligence, normal physique | Under treatment |
| 6 | OTCD | AAs | 12 | 26 | Normal intelligence, normal physique | Under treatment |
| 7 | PAHD | AAs | 12 | 23 | Phe-restricted diet；normal intelligence, normal physique | Under treatment |
| 8 | PAHD | AAs | 8 | 519 | Phe-restricted diet；normal intelligence, normal physique | Under treatment |
| 9 | PAHD | AAs | 8 | 665 | Phe-restricted diet；normal intelligence, normal physique | Under treatment |
| 10 | PAHD | AAs | 12 | 557 | Phe-restricted diet；normal intelligence, normal physique | Under treatment |
| 11 | PAHD | AAs | 12 | 29 | Phe-restricted diet；normal intelligence, normal physique | Under treatment |
| 12 | PAHD | AAs | 16 | N/A | Phe-restricted diet；normal intelligence, normal physique | Under treatment |
| 13 | PAHD | AAs | 14 | N/A | Phe-restricted diet；normal intelligence, normal physique | Under treatment |
| 14 | PAHD | AAs | 14 | 27 | Phe-restricted diet；normal intelligence, normal physique | Under treatment |
| 15 | PAHD | AAs | 13 | N/A | Phe-restricted diet；normal intelligence, normal physique | Under treatment |
| 16 | PAHD | AAs | 12 | 19 | Phe-restricted diet；normal intelligence, normal physique | Under treatment |
| 17 | PAHD | AAs | 10 | 30 | Phe-restricted diet；normal intelligence, normal physique | Under treatment |
| 18 | PAHD | AAs | 14 | 27 | Phe-restricted diet；normal intelligence, normal physique | Under treatment |
| 19 | PAHD | AAs | 12 | N/A | Phe-restricted diet；normal intelligence, normal physique | Under treatment |
| 20 | PAHD | AAs | 16 | N/A | Phe-restricted diet；normal intelligence, normal physique | Under treatment |
| 21 | PAHD | AAs | 11 | N/A | Phe-restricted diet；normal intelligence, normal physique | Under treatment |
| 22 | PAHD | AAs | 13 | 20 | Phe-restricted diet；normal intelligence, normal physique | Under treatment |
| 23 | PAHD | AAs | 16 | 27 | Phe-restricted diet；normal intelligence, normal physique | Under treatment |
| 24 | PAHD | AAs | 14 | 20 | Phe-restricted diet；normal intelligence, normal physique | Under treatment |
| 25 | PAHD | AAs | 11 | 941 | Phe-restricted diet；normal intelligence, normal physique | Under treatment |
| 26 | PAHD | AAs | 15 | 28 | Phe-restricted diet；normal intelligence, normal physique | Under treatment |
| 27 | PAHD | AAs | 15 | N/A | Phe-restricted diet；normal intelligence, normal physique | Under treatment |
| 28 | PAHD | AAs | 13 | N/A | Phe-restricted diet；normal intelligence, normal physique | Under treatment |
| 29 | PAHD | AAs | 19 | 25 | Phe-restricted diet；normal intelligence, normal physique | Under treatment |
| 30 | PAHD | AAs | 11 | 23 | Phe-restricted diet；normal intelligence, normal physique | Under treatment |
| 31 | PAHD | AAs | 16 | 28 | Phe-restricted diet；normal intelligence, normal physique | Under treatment |
| 32 | TYR-II-deficiency | AAs | 11 | 27 | Normal intelligence, normal physique | Under treatment |
| 33 | PAHD | AAs | 10 | 17 | Phe-restricted diet；normal intelligence, normal physique | Under treatment |
| 34 | PAHD | AAs | 14 | 38 | Phe-restricted diet；normal intelligence, normal physique | Under treatment |
| 35 | PAHD | AAs | 13 | 26 | Phe-restricted diet；normal intelligence, normal physique | Under treatment |
| 36 | PAHD | AAs | 10 | 21 | Phe-restricted diet；normal intelligence, normal physique | Under treatment |
| 37 | PAHD | AAs | 9 | 22 | Phe-restricted diet；normal intelligence, normal physique | Under treatment |
| 38 | PAHD | AAs | 11 | 19 | Phe-restricted diet；normal intelligence, normal physique | Under treatment |
| 39 | BH4D | AAs | 20 | 26 | Normal intelligence, normal physique | Under treatment |
| 40 | PAHD | AAs | 10 | 23 | Phe-restricted diet；normal intelligence, normal physique | Under treatment |
| 41 | CIT I-deficiency | AAs | 17 | 22 | Normal intelligence, normal physique | Under treatment |
| 42 | PAHD | AAs | 11 | N/A | Phe-restricted diet；normal intelligence, normal physique | Under treatment |
| 43 | PAHD | AAs | 15 | 29 | Phe-restricted diet；normal intelligence, normal physique | Under treatment |
| 44 | PAHD | AAs | 13 | 65 | Phe-restricted diet；normal intelligence, normal physique | Under treatment |
| 45 | H-MET | AAs | 9 | 42 | Low methionine diet; normal intelligence, normal physique | Under treatment |
| 46 | PAHD | AAs | 15 | 28 | Phe-restricted diet；normal intelligence, normal physique | Under treatment |
| 47 | PAHD | AAs | 11 | 24 | Phe-restricted diet；normal intelligence, normal physique | Under treatment |
| 48 | PAHD | AAs | 10 | 24 | Phe-restricted diet；normal intelligence, normal physique | Under treatment |
| 49 | PAHD | AAs | 18 | N/A | Phe-restricted diet；normal intelligence, normal physique | Under treatment |
| 50 | PAHD | AAs | 12 | 25 | Phe-restricted diet；normal intelligence, normal physique | Under treatment |
| 51 | H-MET | AAs | 12 | 26 | Low methionine diet; normal intelligence, normal physique | Under treatment |
| 52 | PAHD | AAs | 30 | 42 | Phe-restricted diet；normal intelligence, normal physique | Under treatment |
| 53 | PAHD | AAs | 12 | 46 | Phe-restricted diet；normal intelligence, normal physique | Under treatment |
| 54 | PAHD | AAs | 13 | 25 | Phe-restricted diet；normal intelligence, normal physique | Under treatment |
| 55 | PAHD | AAs | 15 | 42 | Phe-restricted diet；normal intelligence, normal physique | Under treatment |
| 56 | PAHD | AAs | 30 | 218 | Phe-restricted diet；normal intelligence, normal physique | Under treatment |
| 57 | CACTD | FAODs | 13 | 24 | Recurrent fever; poor response; hypoketotic hypoglycemia; liver injury; die at 1 year 9 months of age | Die |
| 58 | CPT I-deficiency | FAODs | 64 | 77 | Normal intelligence, normal physique | Under treatment |
| 59 | CUD | FAODs | 15 | 27 | Normal intelligence, normal physique | Under treatment |
| 60 | CUD | FAODs | 18 | 29 | Normal intelligence, normal physique | Under treatment |
| 61 | CUD | FAODs | 13 | 27 | Normal intelligence, normal physique | Under treatment |
| 62 | CUD | FAODs | 10 | 27 | L-carnitine supplementation; normal intelligence, normal physiqu | Under treatment |
| 63 | MCADD | FAODs | 21 | 39 | Normal intelligence, normal physique | Under treatment |
| 64 | SCAD | FAODs | 19 | 30 | L-carnitine supplementation; normal intelligence, normal physiqu | Under treatment |
| 65 | SCAD | FAODs | 14 | 26 | L-carnitine supplementation; normal intelligence, normal physiqu | Under treatment |
| 66 | SCAD | FAODs | 11 | 32 | L-carnitine supplementation; normal intelligence, normal physiqu | Under treatment |
| 67 | SCAD | FAODs | 9 | 24 | L-carnitine supplementation; normal intelligence, normal physiqu | Under treatment |
| 68 | SCAD | FAODs | 10 | 29 | L-carnitine supplementation; normal intelligence, normal physiqu | Under treatment |
| 69 | SCAD | FAODs | 10 | 21 | L-carnitine supplementation; normal intelligence, normal physiqu | Under treatment |
| 70 | SCAD | FAODs | 16 | 29 | L-carnitine supplementation; normal intelligence, normal physiqu | Under treatment |
| 71 | VLCADD | FAODs | 28 | 38 | Milk powder product fortified with medium-chain triglyceride and restricted with galactose; normal intelligence, normal physique | Under treatment |
| 72 | CUD | FAODs | 10 | 20 | Normal intelligence, normal physique | Under treatment |
| 73 | MCADD | FAODs | 14 | 27 | L-carnitine supplementation; normal intelligence, normal physiqu | Under treatment |
| 74 | SCAD | FAODs | 11 | 24 | L-carnitine supplementation; normal intelligence, normal physiqu | Under treatment |
| 75 | CUD | FAODs | 10 | 23 | Normal intelligence, normal physique | Under treatment |
| 76 | SCAD | FAODs | 15 | 27 | L-carnitine supplementation; normal intelligence, normal physiqu | Under treatment |
| 77 | SCAD | FAODs | 25 | 44 | L-carnitine supplementation; normal intelligence, normal physiqu | Under treatment |
| 78 | SCAD | FAODs | 15 | 26 | L-carnitine supplementation; normal intelligence, normal physiqu | Under treatment |
| 79 | 2-MBG--deficiency | OAs | 18 | N/A | Normal intelligence, normal physique | Under treatment |
| 80 | 3MCCD | OAs | 22 | 397 | Normal intelligence, normal physique | Under treatment |
| 81 | 3MCCD | OAs | 12 | 24 | Normal intelligence, normal physique | Under treatment |
| 82 | 3MCCD | OAs | 16 | 29 | Normal intelligence, normal physique | Under treatment |
| 83 | GA II-deficiency | OAs | 11 | 23 | L-carnitine supplementation; normal intelligence, normal physiqu | Under treatment |
| 84 | IBDD | OAs | 14 | 31 | Normal intelligence, normal physique | Under treatment |
| 85 | IBDD | OAs | 16 | 29 | Normal intelligence, normal physique | Under treatment |
| 86 | MAHCC-deficiency | OAs | 16 | 120 | Died 6 months after birth | Die |
| 87 | MAHCC-deficiency | OAs | 18 | 43 | L-carnitine,VB12 and betaine supplementation; normal intelligence, normal physique | Under treatment |
| 88 | MAHCC-deficiency | OAs | 18 | 31 | N/A | Lost to follow-up |
| 89 | MMA-MUTD | OAs | 14 | 26 | L-carnitine and VB12 supplementation; normal intelligence, normal physique | Under treatment |
| 90 | MMA-MUTD | OAs | 15 | 27 | L-carnitine supplementation; normal intelligence, hypotonia and developmental delays | Under treatment |
| 91 | MMA-MUTD | OAs | 9 | 23 | N/A | Lost to follow-up |
| 92 | PA | OAs | 16 | 29 | N/A | Lost to follow-up |
| 93 | 3MCCD | OAs | 7 | 30 | Normal intelligence, normal physique | Under treatment |
| 94 | MAHCC-deficiency | OAs | 11 | 23 | L-carnitine,VB12 and betaine supplementation; normal intelligence, normal physique | Under treatment |
| 95 | GA I-deficiency | OAs | 15 | 28 | Normal intelligence, normal physique | Under treatment |
| 96 | 3MCCD | OAs | 11 | 390 | Normal intelligence, normal physique | Under treatment |
| 97 | MAHCC-deficiency | OAs | 9 | 21 | L-carnitine,VB12 and betaine supplementation; normal intelligence, normal physique | Under treatment |
| 98 | IBDD | OAs | 16 | 30 | Normal intelligence, normal physique | Under treatment |
| 99 | MAHCC-deficiency | OAs | 14 | 27 | L-carnitine,VB12 and betaine supplementation; normal intelligence, normal physique | Under treatment |
| 100 | 3MCCD | OAs | 12 | 22 | Normal intelligence, normal physique | Under treatment |
