# Supplementary material for: Expanded newborn screening for inherited metabolic disorders by tandem mass spectrometry in a northern Chinese population
Source: Front Genet. 2022 Sep 30;13:801447. doi: 10.3389/fgene.2022.801447 (PMC9562093; doi:10.3389/fgene.2022.801447)
Supplement: Supplementary file 3 [file Table3.DOCX]

**Table S2** Molecular diagnosis of 22 patients with organic acid disorders

| No. | Gender | Disorders | Affected Gene | Allele 1 | Allele 2 |
| --- | --- | --- | --- | --- | --- |
| 1 | Female | 2-methylbutyrylglycinuria deficiency (2-MBG- deficiency) | *ACADSB* | *c.18G>A* | *c.43-6570C>T* |
| 2 | Female | 3-methylcrotonyl CoA carboxylase dedicency (3-MCCD) | *MCCC2* | *c.166C>T* | *c.363del* |
| 3 | Female | 3-methylcrotonyl CoA carboxylase dedicency (3-MCCD) | *MCCC2* | *c.281+1G>T* | *c.595G>T* |
|  |  |  | *ACADS* | *c.164C>T* |  |
| 4 | Female | Isobutyryl-CoA dehydrogenase deficiency (IBDD) | *ACAD8* | *c.286G>A* | *c.1030A>G* |
|  |  |  | *PAH* | *c.728G>A* |  |
| 5 | Male | Methylmalonic aciduria mut type deficiency (MMA-MUTD) | *MUT* | *c.323G>A* | *c.729_730insTT* |
| 6 | Male | Methylmalonic aciduria mut type deficiency (MMA-MUTD) | *MUT* | *c.323G>A* | *c.1610T>A* |
| 7 | Male | Methylmalonic aciduria and homocystinuria type C (MAHCC-deficiency) | *MMACHC* | *c.349G>C* | *c.440G>A* |
| 8 | Male | Methylmalonic aciduria mut type deficiency (MMA-MUTD) | *MUT* | *c.446A>G* | *c.729_730insTT* |
| 9 | Male | Methylmalonic aciduria and homocystinuria type C (MAHCC-deficiency) | *MMACHC* | *c.482G>A* | *c.609G>A* |
| 10 | Female | Glutaric acidemia type I deficiency (GA I-deficiency) | *GCDH* | *c.523G>A* | *c.1109T>C* |
| 11 | Female | Glutaric acidemia type II deficiency deficiency (GA II-deficiency) | *ETFDH* | *c.524G>A* | *c.1395T>G* |
| 12 | Male | Propionic acidemia (PA) | *PCCB* | *c.605G>A* | *c.1150T>G* |
| 13 | Male | Methylmalonic aciduria and homocystinuria type C (MAHCC-deficiency) | *MMACHC* | *c.609G>A* |  |
| 14 | Female | Methylmalonic aciduria and homocystinuria type C (MAHCC-deficiency) | *MMACHC* | *c.609G>A* | *c.658_660delAAG* |
| 15 | Male | Methylmalonic aciduria and homocystinuria type C (MAHCC-deficiency) | *MMACHC* | *c.609G>A* | *c.80A>G* |
| 16 | Male | Methylmalonic aciduria and homocystinuria type C (MAHCC-deficiency) | *MMACHC* | *c.609G>A* | *c.658_660del* |
| 17 | Female | 3-methylcrotonyl CoA carboxylase dedicency (3-MCCD) | *MCCC1* | *c.639+2T>A* | *c.1894C>T* |
| 18 | Female | Isobutyryl-CoA dehydrogenase deficiency (IBDD) | *ACAD8* | *c.958G>A* | *c.1176G>T* |
| 19 | Male | Isobutyryl-CoA dehydrogenase deficiency (IBDD) | *ACAD8* | *c.1000C>T* | *c.1176G>T* |
| 20 | Male | 3-methylcrotonyl CoA carboxylase dedicency (3-MCCD) | *MCCC2* | *c.1073-6T>A* | *c.1658_1659del* |
|  |  |  | *BTD* | *c.219T>G* |  |
| 21 | Female | 3-methylcrotonyl CoA carboxylase dedicency (3-MCCD) | *MCCC2* | *c.1144_1147delinsTTTT* |  |
|  |  |  | *CLPB* | *c.1559G>A* |  |
| 22 | Male | 3-methylcrotonyl CoA carboxylase dedicency (3-MCCD) | *MCCC1* | *c.1630del* | *c.1894C>T* |
