# Supplementary material for: Expanded newborn screening for inherited metabolic disorders by tandem mass spectrometry in a northern Chinese population
Source: Front Genet. 2022 Sep 30;13:801447. doi: 10.3389/fgene.2022.801447 (PMC9562093; doi:10.3389/fgene.2022.801447)
Supplement: Supplementary file 4 [file Table4.DOCX]

**Table S3** Molecular diagnosis of 22 patients with fatty acid oxidation disorders

| No. | Gender | Disorders | Affected Gene | Allele 1 | Allele 2 |
| --- | --- | --- | --- | --- | --- |
| 1 | Male | Carnitine-acylcarnitine translocase deficiency (CACTD) | *SLC25A20* | *c.1A>T* | *c.199-10T>G* |
| 2 | Female | Carnitine uptake defect (CUD) | *SLC22A5* | *c.53A>T* | *c.1400C>G* |
|  |  |  | *PCCA* | *c.1882C>T* |  |
| 3 | Male | Medium-chain acyl-CoA dehydrogenase deficiency (MCADD) | *ACADM* | *c.91C>T* | *c.985A>G* |
| 4 | Female | Short-chain acyl-CoA dehydrogenase deficiency (SCAD) | *ACADS* | *c.162dup* | *c.1130C>T* |
| 5 | Male | Short-chain acyl-CoA dehydrogenase deficiency (SCAD) | *ACADS* | *c.164C>T* | *c.1031A>G* |
| 6 | Male | Short-chain acyl-CoA dehydrogenase deficiency (SCAD) | *ACADS* | *c.164C>T* | *c.1031A>G* |
| 7 | Male | Short-chain acyl-CoA dehydrogenase deficiency (SCAD) | *ACADS* | *c.164C>T* | *c.1031A>G* |
| 8 | Male | Short-chain acyl-CoA dehydrogenase deficiency (SCAD) | *ACADS* | *c.164C>T* | *c.1031A>G* |
| 9 | Male | Short-chain acyl-CoA dehydrogenase deficiency (SCAD) | *ACADS* | *c.164C>T* | *c.307G>A* |
| 10 | Female | Short-chain acyl-CoA dehydrogenase deficiency (SCAD) | *ACADS* | *c.164C>T* |  |
|  |  |  | *PTS* | *c.169G>A* |  |
| 11 | Female | Short-chain acyl-CoA dehydrogenase deficiency (SCAD) | *ACADS* | *c.322G>A* |  |
| 12 | Female | Medium-chain acyl-CoA dehydrogenase deficiency (MCADD) | *ACADM* | *c.232A>G* | *c.449_452del* |
| 13 | Female | Short-chain acyl-CoA dehydrogenase deficiency (SCAD) | *ACADS* | *c.360+32G>A* | *c.1031A>G* |
|  |  |  | *ETFDH* | *c.389A>T* |  |
| 14 | Male | Carnitine uptake defect (CUD) | *SLC22A5* | *c.428C>T* | *c.1400C>G* |
| 15 | Male | Carnitine uptake defect (CUD) | *SLC22A5* | *c.428C>T* | *c.1400C>G* |
| 16 | Female | Carnitine palmitoyl transferase-I deficiency (CPT-I-deficiency) | *PAH* | *c.473G>A* |  |
| 17 | Male | Very long-chain acyl-CoA dehydrogenase deficiency (VLCADD) | *ACADVL* | *c.652G>A* | *c.1367G>A* |
| 18 | Male | Short-chain acyl-CoA dehydrogenase deficiency (SCAD) | *ACADSB* | *c.655G>A* | *c.848A>G* |
|  |  |  | *ACADM* | *c.1085G>A* |  |
| 19 | Male | Carnitine uptake defect (CUD) | *SLC22A5* | *c.1265dupC* | *c.1400C>G* |
| 20 | Female | Short-chain acyl-CoA dehydrogenase deficiency (SCAD) | *ACADS* | *c.1031A>G* | *c.1130C>T* |
| 21 | Male | Carnitine uptake defect (CUD) | *SLC22A5* | *c.1400C>G* | *c.1265dup* |
| 22 | Male | Carnitine uptake defect (CUD) | *SLC22A5* | *c.1400C>G* | *c.1265dup* |
