# Supplementary material for: Expanded newborn screening for inherited metabolic disorders by tandem mass spectrometry in a northern Chinese population
Source: Front Genet. 2022 Sep 30;13:801447. doi: 10.3389/fgene.2022.801447 (PMC9562093; doi:10.3389/fgene.2022.801447)
Supplement: Supplementary file 5 [file Table6.DOCX]

Dear Editor and Reviewers,

Thank you very much for considering a revision of our manuscript.

We are grateful to the reviewers for carefully reviewing and constructively commenting on our work. We have addressed all their comments and incorporated their helpful suggestions into the revised manuscript. Our response to the comments is enclosed.

Sincerely yours,

Hong Zhang

Enclosed: responses

# To REVIEWER 1

**Question 1:** Introduction: Not all the statements are adequately referenced.

**Answer:** We carefully re-wrote and added some content and pertinent literature in Introduction of the manuscript.

**Question 2:** Methods: The description of the process of NBS is not enough detailed. I suggest that a Flowchart is added, depicting all the phases of NBS in more detail. Could you add the full list of genetic panel used? Can you add the details regarding the process of confirmatory diagnostic process.

**Answer:** We revised the Materials and methods part and added the flowchart of newborn screening.

**Question 3:** Results: Would be good to present the results also with additional data on the levels of metabolites in all of the individuals with positive NBS results, to allow better genotype-phenotype correlations assessments.

**Answer:** Thank you for your advice. The table and legend have been amended. We believe that the revised form will now well prepare readers.

**Question 4:** Discussion: Regarding the incidences of IEMs with similar studies it depends on both he characteristics of population and also regarding the inclusion criteria (one comparable report is following:10.1016/j.clinbiochem.2017.10.016, which could help to better address this issue). Could you present and compare it in the Table also from the perspective of population/methodology. In addition, not all the disorders screened are considered they should be screened (e.g. 3-MCC, SCADD) - could you please add this notion and further discuss it.

In addition, especially for non-Chinese readers and for the global perspective, it would be good to put your study more clearly in the wider, pan-Chinese context of expanded NBS programs.

**Question 3:**

1) We appreciate the reviewer’s insightful suggestion and interesting point. However, the universal NBS has been recommended by the Chinese regional governments. Nevertheless, we recognize this limitation should be mentioned in the paper, so we added the following sentence in Discussions section: With regard to M/SMS screening efficiency, like most newborn screening centers in China, we did not use any second tier testing so that we had a high positive rate of initial screening.
2) We followed the guidelines of NBS in China and did not conduct selective screening. We have listed all screening items in the Materials and methods section.
3) The data of NBS in other regions of China and other countries have been added in the revised manuscript of Discussions.

**Question 5:**

-English language should be improved somewhat, many unusual formulations (e.g. "never uncommon")

-Once abbreviation is introduced, it should be consistently used

-MCDA - should be MCAD?

-Please find appropriate references for all the statements.

**Answer:**

1) Thank you for your suggestion. Modifications have been made one by one. The wording errors in the translation of Chinese into English have been revised. We invited experts whose native language is English to make the revision.
2) We checked the consistency of all our abbreviations and spell out the full name of abbreviation at the first time of the usage.
3) This has been modified.
4) We added the pertinent literature for all the statements.

# To REVIEWER 3

**Question 1:** This article reveals the incidence of AAs, OAs, and FAODs using ENBS in Suqian city, northern China. Due to the large territory of China, there are regional differences even within the same China, and this report is useful information for researchers who involved in ENBS in northern China. In other words, this result is beneficial for only a small number of researchers.

**Answer:** We appreciate the reviewer’s insightful suggestion and interesting point. However, our practice has implications for newborn screening in other regions. At the same time, the data of NBS in other regions of China and other countries have been added in the revised manuscript of Discussions.

**Question 2:** There are also two major problems in this report. One is the description of the results or counting the variants. The authors listed the heterozygous variant (carrier) that were accidentally detected by NGS, but this counting is meaningless and should be deleted altogether. For example, a heterozygous variant in PCCA gene should have no effect on the condition of a patient with CUD. However, one case, a patient with MCAD deficiency has a compound heterozygous mutation in SBCAD, is unique. The diagnosis of this patient should be considered.

**Answer:** This was an error and the error has been corrected. The detection results see the attachment.

**Question 3:** The other problem is that the results and considerations are poor. For example, the first paragraph of this discussion section is almost general information but not discussion. The authors should more consider the unique points in Suqian and the difference from other regions of China and other Asian countries. In addition, there are many small careless mistakes as shown in the minor comments. Although the English expression is grammatically enough for me (I’m not a native speaker), a native expert should proofread this draft.

**Answer:**

1) We have transferred the first paragraph of Discussion section to Introduction of the manuscript.
2) The discussions of other regions of China and other Asian countries have been added in the revised manuscript.
3) The wording errors in the translation of Chinese into English have been revised. We invited experts whose native language is English to make the revision.

**Question 4:** Gene name should be described as italic type. Add a comma to numbers over 1,000

**Answer:** All gene names have been described as italic type. All numbers over 1,000 have been added a comma.

**Question 5:**

- Spell out CUD.

- What did “The level of amino acid disorders, …disorders…”mean? Did the authors measure the serum concentrations of amino acids, organic acids, and acylcarnitines?

- In same phrase, the authors mentioned that some kinds of levels were measured in serum, but did not describe the details in the material and methods section.

**Answer:**

1) This has been modified.
2) Thank you for pointing out. We have revised the abstract and added the details in the material and methods section.
3) Thank you for pointing out. We have revised the abstract and added the details in the material and methods section.

**Question 6:**

Running title

NS should be changed to NBS.

**Answer:** This has been modified.

**Question 7:**

Keyword and draft

second generation sequencing (next generation sequencing, NGS) should be unified into NGS (2nd generation sequencing).

**Answer:**

Thank you for pointing out. We did not uniformly modify “second generation sequencing (next generation sequencing, NGS)” to “NGS (2nd generation sequencing)” as “2nd generation sequencing” cannot be queried in NIBI MeSH, but "next generation sequencing" can be queried.

**Question 8:**

Introduction

At page 5, line25th, what region is the incidence? (whole China?)

At page 6, line 2nd, “Second-“should be lowercase.

**Answer:**

1) The wording has been modified.
2) The wording has been modified.

**Question 9:**

Materials and methods

List all target diseases of ENBS in Suqian city.

At page6, line 14 and 15th, spell out HPLC and AAs (not AAS)

At page 6, line 21th or page 7, line 15th, same phrase was used, please deleted an either.

At page 7, line 12th, “NKH” is maybe non-ketotic “hypoglycemia”, isn’t it?

**Answer:**

1) We have now added a description of ENBS in Suqian city.
2) They have been modified.
3) Thank you for pointing out. We have deleted the latter.
4) The wording has been modified.

**Question 10:**

Results

At page 7, line 27, PPV should be calculated with 4,069 positive cases in the first screening as the denominator

At page 8, line 7th, “45/56” is correct.

At not only page 8, line 8th, but also other parts, spell out BH4D, HCY, etc. although abbreviation was listed up.

At page 8, line 15th, Table “1S” is correct.

At page 8, line 15th, if there were no siblings with PAHD, the variant frequency of 45 patients with PAHD should be calculated with 90 alleles (not 110) as denominator.

At page 8, the font of “CD” and “H-Met” is strange.

At page 8, line 17th, three patients with CD have a “compound heterozygous (not simple “heterozygous”)” mutation in SLC25A13. The author must understand the difference between compound heterozygote and heterozygote.

At page 8, line 19th, “One patient has a “compound” heterozygous・・・“ is correct.

**Answer:**

1) This has been modified.
2) This has been modified.
3) We checked the consistency of all our abbreviations and spell out the full name of abbreviation at the first time of the usage.
4) They have been modified. We have been modified the description of this paragraph.
5) These fonts have been modified.
6) Thank you for pointing out. We have modified the wording of heterozygous in the manuscript.
7) This has been modified.

**Question 11:**

Results section of “Organic acid disorders”

At page 8, line 27, please recount the number of variants in MCCC1 and MCCC2.

At page 8, line 30, the frequency of *c.609G>A* is 41.67% (5/12).

At page 9, line 1st, the most common allele frequency of MUT is each 33.33% (not 66.67%).

At page 9, line 2nd, the remaining patients did not have homozygous mutation.

**Answer:**

We apologize for the erroneous data on page 8-9. They have been modified. We modified this paragraph. (See the main text in revised manuscript)

**Question 12:**

Results section of “Fatty acid disorders”

Use lowercases in the first letter of disease name.

VLCAD, MCAD, CPT, and CACT are enzyme name, not disease name. For example, change VLCAD to VLCADD or VLCAD deficiency.

Medium chain acyl-CoA dehydrogenase is generally abbreviated as MCAD. Please correct MCDA in not only this section but also other sections including Table 1 and Table 3S.

Although a patients had a compound heterozygous variant in ACADSB. He/she was diagnosed with MCADD. Is this true? I think that this patient should be diagnosed as SBCAD deficiency. The authors should describe the details of diagnosis.

At page 9, line 19, when allele frequency is calculated, don’t unify different genes. For example, the frequency of c.1031A>G in ACADS is 30% (6/20). Besides, the variant of c.1031A>G was described twice (and the allele frequency was different).

**Answer:**

1) This has been modified accordingly, and we have done so throughout the manuscript.
2) They have been modified.
3) They have been modified.
4) This was an error and the error has been corrected. The detection results see the attachment.
5) Thank you for pointing out. We have reanalyzed the data and modified the wording of this paragraph.

**Question 13:**

Discussion

Because the first paragraph is all general information, please transfer to background, or delete it.

The first half of second paragraph is also poor and almost same to results. The last half is just a quote of other articles, and is not debated.

This conclusion is inappropriate. Although MS/MS is surely a powerful tool, this fact is general, and the authors’ result could not indicate this conclusion. For example, this positive rate of the first screening (1.99%) was likely to be higher than other reports. NGS is also powerful and enhance the accuracy of diagnosis, but cannot directly reduce the false positive rate of the first screening.

**Answer:**

1) We have transferred the first paragraph of Discussion section to Introduction of the manuscript.
2) Discussion section has been modified and added the detailed discussion.

**Question 14:**

Abbreviations

Aceylcarnitine → acetylcarnitine

**Answer:**

They have been modified.

**Question 15:**

Table 1

The order of OAs and FAODs should be exchanged. Moreover, please list IMDs that was included in the ENBS panels but not found.

Table 2 Spell out Phe, Tyr, etc in the legends; Frequency is not correct (calculated as denominator of 59).

When the values were over double digits, please use one digit after decimal point.

What is the reference range of CIT-I?

Table 4

The value of C5 is not necessary in MCADD if the patient really have MCADD.

Table S1～S3

Please sort the patients list in the order of gene position (c.DNA) numbers, and the variant with smaller gene position number should be placed in the left (the side of “allele 1”)

**Answer:**

1) Table 1 has been modified as suggested.
2) As suggested, the abbreviations have been spelled out in the legends; we have now combined table1 to table4, and fixed the error in figure 2; we used two-digit numbers in order to further unify the standard; the reference range of CIT-I has been added.
3) The value of C5 has been deleted.
4) Table S1-Table S3 has been revised a suggested.

# To REVIEWER 4

**Question 1:** The article has no clear object，the object of abstract is inconsistent with that of introduction.

The present study aimed to evaluate the incidence, disease spectrum, follow-up results, and genetic characteristics of inherited metabolic disorders (IMDs) of newborns in Suqian city or to explore the application value of MS/MS technologies and second-generation DNA sequencing in NBS?

**Answer:** Thank you for pointing out this issue. We have rephrased first two sentences of the Abstract following your suggestion.

**Question 2:** Abstract - The contents of the serum to be tested were incorrectly stated.

**Answer:** There is no description about the contents of the serum in the Abstract. Other data have been checked and modified accordingly.

**Question 3:** Introduction - The introduction is too brief, define the problem clearly and detailly.

**Answer:** We carefully re-wrote and added some content in Introduction of the manuscript.

**Question 4:** Methods and results are lack of the part of follow-up.

**Answer:** The follow-up have been added in Methods and results.

**Question 5:** Method (Newborn screening)– The information of instrument and reagent were vague, company details, location missing.

**Answer:** The information of instrument and reagent have been added.

**Question 6:** Method and Results – Details of routine biochemical have not been provided in the method and result section.

**Answer:**

We appreciate the reviewer’s insightful suggestion and interesting point. But we are actively looking for collaboration to address this issue, and hopefully will provide information in the future. But in any case, the corresponding description was added in the Discussion.

**Question 7:** change to the pie chart to show the disease spectrum and distribution of IMDs

**Answer:** The disease spectrum and distribution of IMDs have been changed to the pie chart

**Question 8:** Discussion - Lack of discussion of OAs and FAODs

**Answer:** The discussion of OAs and FAODs has been added in the Discussion.
